# RFX5 promotes the progression of hepatocellular carcinoma through transcriptional activation of KDM4A

**DOI:** 10.1038/s41598-020-71403-1

**Published:** 2020-09-03

**Authors:** Dong-Bo Chen, Xing-Wang Xie, Yang-Jing Zhao, Xue-Yan Wang, Wei-Jia Liao, Pu Chen, Kang-Jian Deng, Ran Fei, Wan-Ying Qin, Jiang-Hua Wang, Xu Wu, Qi-Xiang Shao, Lai Wei, Hong-Song Chen

**Affiliations:** 1 Peking University People’s Hospital, Peking University Hepatology Institute, Beijing Key Laboratory of Hepatitis C and Immunotherapy for Liver Disease, Beijing, 100044 China; 2 Department of Immunology, and the Key Laboratory of Laboratory Medicine of Jiangsu Province, School of Medicine, Jiangsu University, Zhenjiang, 212013 Jiangsu China; 3 Laboratory of Hepatobiliary and Pancreatic Surgery, Affiliated Hospital of Guilin Medical University, Guilin, China; 4 Center of Excellence, Becton Dickinson Biosciences, China Central Place, Beijing, 100176 China

**Keywords:** Cancer, Gastrointestinal cancer, Oncogenes, Tumour biomarkers, Cancer, Oncology, Risk factors, Gastroenterology, Hepatology, Prognostic markers, Gastrointestinal cancer, Molecular medicine

## Abstract

Regulatory factor X-5 (RFX5) represents a key transcription regulator of MHCII gene expression in the immune system. This study aims to explore the molecular mechanisms and biological significance of RFX5. Firstly, by analyzing ENCODE chromatin immunoprecipitation (ChIP)-seq in HepG2 and TCGA RNA-seq data, we discovered lysine-specific demethylase 4A (KDM4A), also named JMJD2A, to be a major downstream target gene of RFX5. Moreover, RFX5 was verified to bind directly to the KDM4A’s promoter region and sequentially promoted its transcription determined by the ChIP-PCR assay and luciferase assay. In addition, RFX5-dependent regulation of KDM4A was demonstrated in HCC. Compared with adjacent non-tumor tissues, the expression levels of KDM4A were significantly raised in HCC tumor tissues. Notably, elevated levels of KDM4A were strongly correlated with HCC patient prognosis. Functionally, KDM4A overexpression largely rescued the growth inhibitory effects of RFX5 deletion, highlighting KDM4A as a downstream effector of RFX5. Mechanistically, the RFX5-KDM4A pathway promoted the progression of the cell cycle from G0/G1 to S phase and was protective against cell apoptosis through regulation of p53 and its downstream genes in HCC. In conclusion, RFX5 could promote HCC progression via transcriptionally activating KDM4A expression.

## Introduction

Hepatocellular carcinoma (HCC) is the fifth most commonly diagnosed and the second most lethal neoplasm in men worldwide. In 2018 alone, it was identified in an estimated 841,080 newly diagnosed HCC patients and 781,631 HCC-related cancer deaths^1^. Although target therapy with sorafenib, lenvatinib, regorafenib and anti-PD1 antibody have achieved better treatment response in certain HCC patients, HCC in its advanced stages is highly lethal in a large proportion of patients^2^. Therefore, treatment strategies for HCC are in dire need for more effective molecular therapeutic targets.

One of the typical molecular mechanisms of cancer is the accumulation of genetic and epigenetic alterations which eventually disrupts cellular pathways, culminated in uncontrolled cellular growth^3^. Intriguingly, a study demonstrated that more than half of its cohort were found to have amplified levels of the 1q21-44 loci on chromosomes, suggesting that this genetic location may house a number of major oncogenes that act as drivers of HCC development, such as CHD1L (1q21.1)^4^, PYCR2 (1q42.12)^5^ and BCL9 (1q21.2)^6^. Furthermore, analysis of genomics data derived from the TCGA Liver hepatocellular carcinoma (LIHC) revealed regulatory factor X-5 (RFX5), located in the chromosome 1q21 loci, have aberrantly raised expressions in HCC patients^7^. A functional study demonstrated that HCC cell colony formation and subcutaneous tumor growth were dependent on RFX5 overexpression. The converse was seen when RFX5 was repressed^7^. These findings suggested that RFX5 might function as a tumor driver gene in HCC.

The RFX5 gene marked by its dimerization motifs and highly conserved DNA-binding domain (DBD) has been unveiled to regulate MHCII gene transcription with the other three factors: CIITA, RFXANK and RFXAP^8,9^. However, RFX5 overexpression in HCC cell lines and tumor tissues failed to provoke a tangible response in MHCII expression, suggesting that other, non-MHCII target genes may be involved in HCC progression^10^. Through systematic identification of RFX5’s transcriptional target genes determined by analyzing RNA-seq data from the TCGA LIHC dataset, this study reveals that RFX5 might also be involved in repair of DNA damage, progression of the cell cycle and proliferation pathways. Intriguingly, RFX5 was protective against HCC-derived cell apoptosis by regulating tyrosine 3-monooxygenase/tryptophan 5-monooxygenase activation protein theta (YWHAQ) transcription^7^. Though YWHAQ appeared to be the downstream target of RFX5, overexpression YWHAQ only partly reversed the growth inhibitory effects mediated by RFX5 deletion in HepG2 cells. These findings implied that RFX5 might promote HCC progression by regulating other target genes, not only YWHAQ.

Therefore, the current investigation aims to determine downstream target genes of RFX5 and explore the underlying pathways of RFX5-dependent development of HCC.

## Results

### RFX5 overexpression is an independent prognostic factor for the survival of patients with advanced HCC

**Figure 1:**
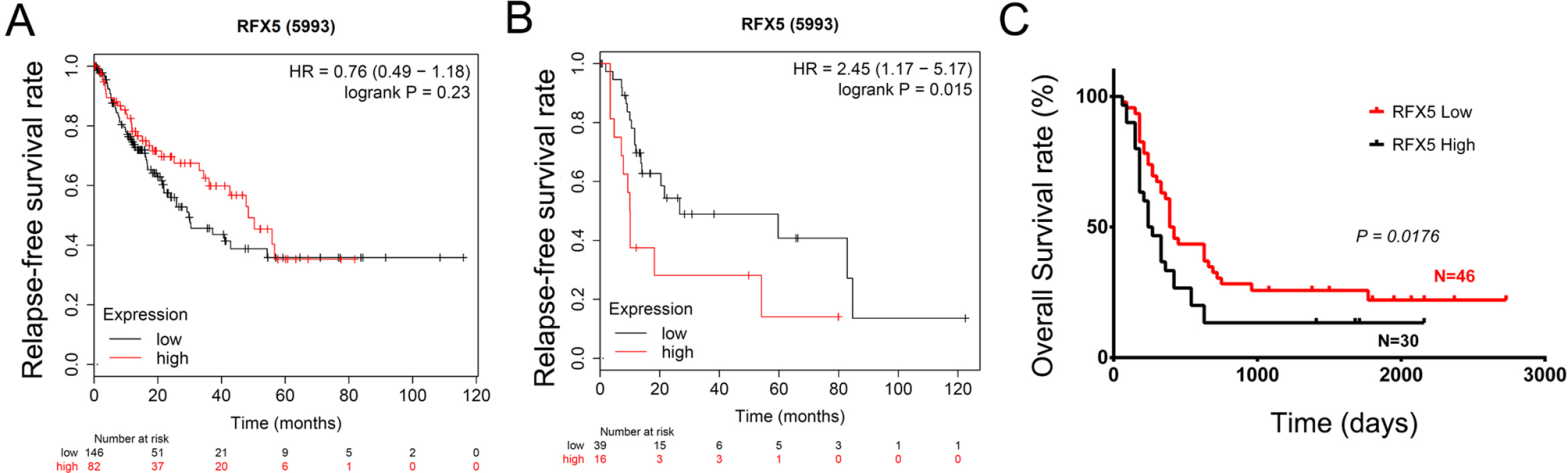
RFX5 overexpression is positively associated with poor prognosis of patients with advanced HCC. (**A**) The correlation of RFX5 mRNA expression level with RFT in patients with early and intermediate-stage HCC determined by analyzing RNAseq data from the TCGA LIHC dataset. (**B**) The correlation of RFX5 mRNA expression level with RFT in patients with advanced stage HCC determined by analyzing RNAseq from the TCGA LIHC dataset. (**C**) The correlation of RFX5 protein expression with OST in patients with advanced stage HCC determined by IHC analysis.

By analyzing the TCGA LIHC dataset, the RFX5 mRNA expression was significantly associated with the prognosis of patients with advanced HCC (Fig. 1B). Patients with a high mRNA expression of RFX5 may have a shorter relapse-free survival time compared with patients with a low RFX5 (P = 0.015). However, there was no relationship between the RFX5 expression and the prognosis of patients with early and intermediate-stage HCC (Fig. 1A). Consistently, Kaplan–Meier survival analysis showed high expression of RFX5 proteins stained by IHC in primary HCC tumor tissue was associated with poor overall survival (P = 0.0176) of patients with advanced HCC in Guilin cohort (Fig. 1C).

### RFX5 binds the promoter region of KDM4A in HCC

Through analysis of ENCODE-project derived database regarding RFX5 ChIP-seq data in HepG2 cells, we found that RFX5 could bind to a large group of genes. To identify primary transcriptional target genes of RFX5 in HCC, we firstly identified the top 1,000 RFX5 binding sites in HepG2 cell, and then mapped these binding sites to protein-coding genes. 646 genes with strong RFX5 binding signals in the promoter region were enrolled for the subsequent integrating data analysis. The mRNA expression level of RFX5 and 646 candidate RFX5 downstream genes, determined by analyzing RNA-seq data from TCGA LIHC dataset, were subjected to correlation analysis.

**Figure 2:**
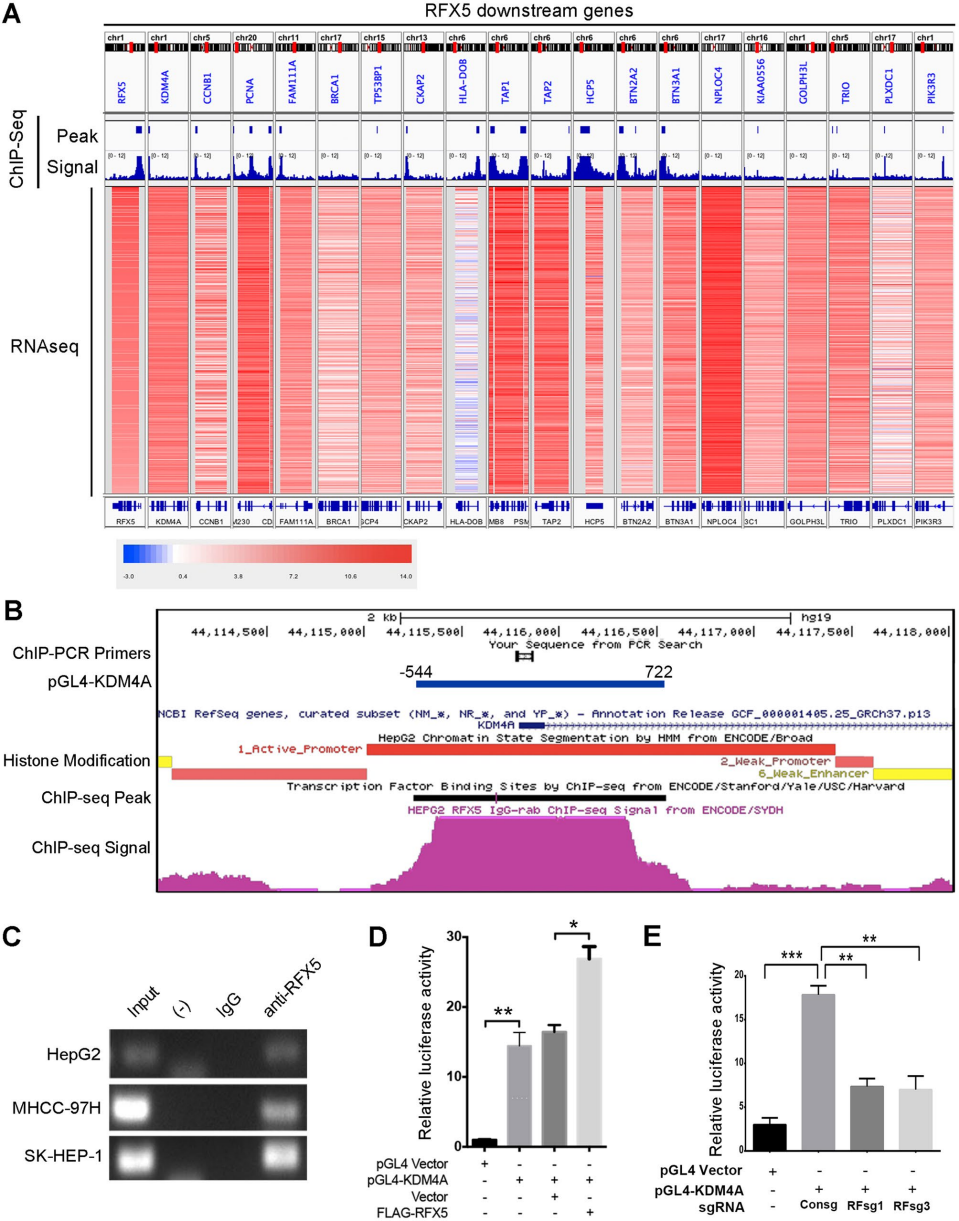
RFX5 binds to the promoter region and activates transcription of the KDM4A genes in HCC. (**A**) Candidate RFX5 target genes determined by analyzing the RFX5 ChIP-seq binding peak and signal of HepG2 cells from ECODE dataset and RNA-seq data from TCGA LIHC dataset. (**B**) The ChIP-seq binding peak and signal of RFX5 in KDM4A promoter regions. The histone modification status and the amplification region of ChIP-PCR were also indicated. (**C**) The immunoprecipitated DNA fragments produced using anti-RFX5 antibody underwent PCR analysis of KDM4A promoter region in HCC cells. (**D**) The transcriptional activity of KDM4A promoter reporter constructs (pGL4-KDM4A) was measured in MHCC-97H utilizing relative luciferase activity (RLA), which was the ratio of firefly to Renilla luciferase activities. The effect of modulation of RFX5 expression on KDM4A transcriptional activity was determined by co-transfection of pGL4-KDM4A and FLAG-RFX5 in MHCC-97H cells. (**E**) The effect of RFX5 expression on the transcriptional activity of KDM4A was identified through co-transfection of sgRNAs targeting RFX5 (RFsg1 or RFsg3) and pGL4-KDM4A in MHCC-97H cells. *P < 0.05, **P < 0.01, ***P < 0.001.

Among genes which both had a strong binding signal of RFX5 in the promoter region and were closely correlated with RFX5 in RNA expression level (Fig. 2A), KDM4A had engrossed our attention due to its reported role in several cancers^12^, including HCC^13^. Hence, we decided to focus on the regulation of RFX5 on KDM4A expression in HCC cells.

The ChIP-seq data in HepG2 cells exhibited a strong binding peak signal of RFX5 in the KDM4A promoter location (Fig. 2B). We then performed ChIP assay in HepG2, MHCC-97H and SK-HEP-1 cells with identical RFX5 antibody which was utilized in the ENCODE project. Immunoprecipitated DNA fragments produced by anti-RFX5 were subjected to PCR assay with primers that can detect the RFX5 binding peak site in the KDM4A promoter region which was determined by ChIP-seq. Consistent with ChIP-seq data, KDM4A promoter region was detected by PCR in the elution of anti-RFX5 in SK-HEP-1, MHCC-97H and HepG2 cells, but not in the elution of control IgG (Fig. 2C).

The ChIP-PCR results confirmed the closely binding of RFX5 to the KDM4A promoter region in HCC. In addition, the promoter region (− 544 to + 722) of KDM4A was cloned into a pGL4 vector for a luciferase reporter assay. Overexpression of RFX5 greatly increased the activity of luciferase in MHCC-97H cells transfected with pGL4-KDM4A reporter plasmid (Fig. 2D). However, knockdown of RFX5 by transfection of sgRNAs targeting RFX5 (RFsg1 and RFsg3) significantly inhibited Luciferase activity mediated by pGL4-KDM4A in MHCC-97H cells (Fig. 2E). These data indicated that KDM4A is a transcriptional target of RFX5 in HCC.

### RFX5 is a positive regulator of KDM4A expression in HCC

**Figure 3:**
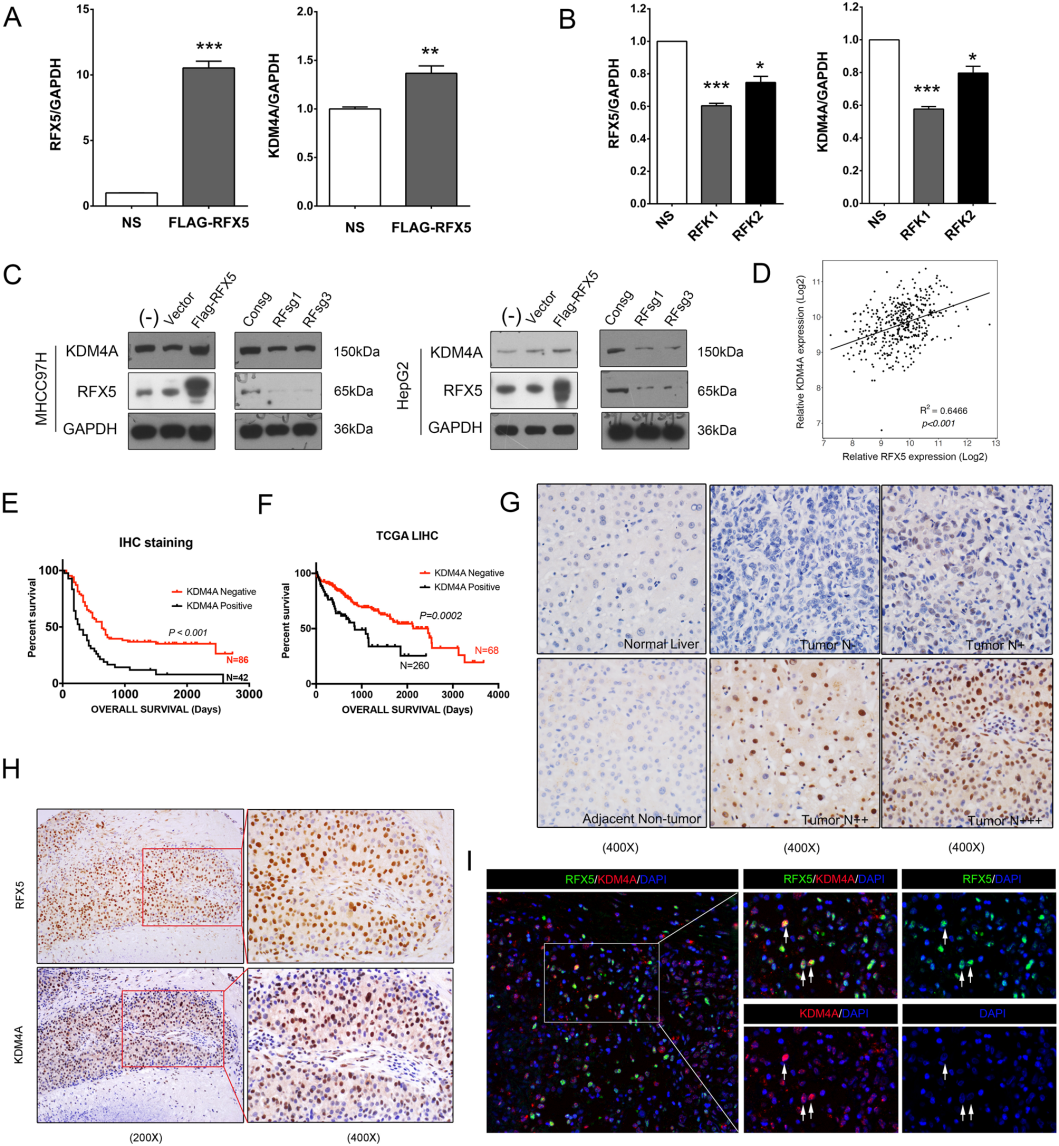
RFX5 is a positive regulator of KDM4A expression in HCC. (**A**) KDM4A expression levels were assessed in FLAG-RFX5-transfected HepG2 cells via QRT-PCR analysis. (**B**) KDM4A expression levels were assessed in shRNA targeting RFX5-transfected HepG2 cells via QRT-PCR analysis. (**C**) KDM4A protein expression levels were determined in MHCC-97H and HepG2 cells transduced with lentiviral FLAG-RFX5 or sgRNA targeting RFX5 via Western blot analysis. (**D**) The correlation between RFX5 and KDM4A mRNA expressions in HCC patients derived from TCGA LIHC dataset. (**E**) The correlation of KDM4A protein expression status determined by IHC analysis with overall survival time (OST). (**F**) The correlation of KDM4A mRNA expression level determined by RNAseq in TCGA LIHC dataset with OST. (**G**) Immunohistochemistry analysis was used to quantify the amount of KDM4A protein in HCC tumor tissues and adjacent non-tumor tissues was determined (N−, less than 5% of tumor cells stained positive; N+, 5–30% of tumor cells stained positive; N++, 31–50% of tumor cells stained positive; N+++, > 51% of tumor cells stained positive.). (**H**) IHC staining of RFX5 and KDM4A in the same cancer nest. (**I**) The co-expression of RFX5 and KDM4A in HCC tissues was determined by the immunofluorescence staining. The white arrows pointed HCC cells which co-expressed RFX5 and KDM4A.

To validate that RFX5 could regulate KDM4A expression in HCC, we directly assessed the impact of RFX5 expression level on the protein and mRNA levels of KDM4A in HCC cells using Western blot and QRT-PCR assays. In HepG2 cells, overexpression of FLAG-RFX5 greatly enhanced the transcription level of KDM4A (Fig. 3A), while knockdown of RFX5 by shRNA significantly decreased the mRNA expression of KDM4A (Fig. 3B). In HepG2 and MHCC-97H cells, overexpression of RFX5 markedly elevated KDM4A protein levels (Fig. 3C). Conversely, knockdown of RFX5 by CRISPR/Cas9 (RFsg1 or RFsg3) significantly downregulated KDM4A protein levels (Fig. 3C). These data indicated that RFX5 could positively regulate KDM4A levels.

As shown in Fig. 3D, we found that KDM4A mRNA levels were strongly associated with those of RFX5 (P < 0.001, R = 0.8041). Next, we also investigated the pattern of KDM4A protein expressions in HCC by performing IHC analysis of the TMA derived from the Guilin cohort. We found that KDM4A was overexpressed in tumor tissue of 42/128 (32.81%) informative HCC cases, but not in non-tumor tissue (Fig. 3G). KDM4A protein located in the cell nucleus showed a heterogenous expression pattern in HCC tumor tissues (Fig. 3G). We then assessed the relationship between RFX5 and KDM4A protein levels in the Guilin cohort. In consistence with mRNA expression, the protein level of KDM4A was significantly associated with that of RFX5 (P = 0.0058) (Supplementary table S1). Notably, the cellular localization and distribution patterns of KDM4A protein were highly consistent with that of RFX5 protein in the continuous sections of the same cancer nest (Fig. 3H). Importantly, RFX5 and KDM4A were co-expressed in HCC cells (Fig. 3I). These results supported that the KDM4A is a downstream target gene of RFX5 in HCC.

Moreover, by analyzing the correlation between KDM4A staining and clinical and biochemical profiles of patients with HCC, we discovered that tumor size was strongly related to an augmented KDM4A expression (P = 0.047) (Supplementary table S1). Intriguingly, Kaplan–Meier survival analysis showed that KDM4A proteins in primary HCC tumor tissue were associated with poor overall survival (P < 0.001) of HCC patients in Guilin cohort (Fig. 3E). Consistently, the KDM4A mRNA expressions levels were strongly associated with HCC patient prognosis (data derived from the TCGA LIHC dataset, P < 0.001, Fig. 3F). High expression of KDM4A appeared to be predictive of poor survival.

### RFX5 promotes tumor growth in a KDM4A-dependent way in HCC

**Figure 4:**
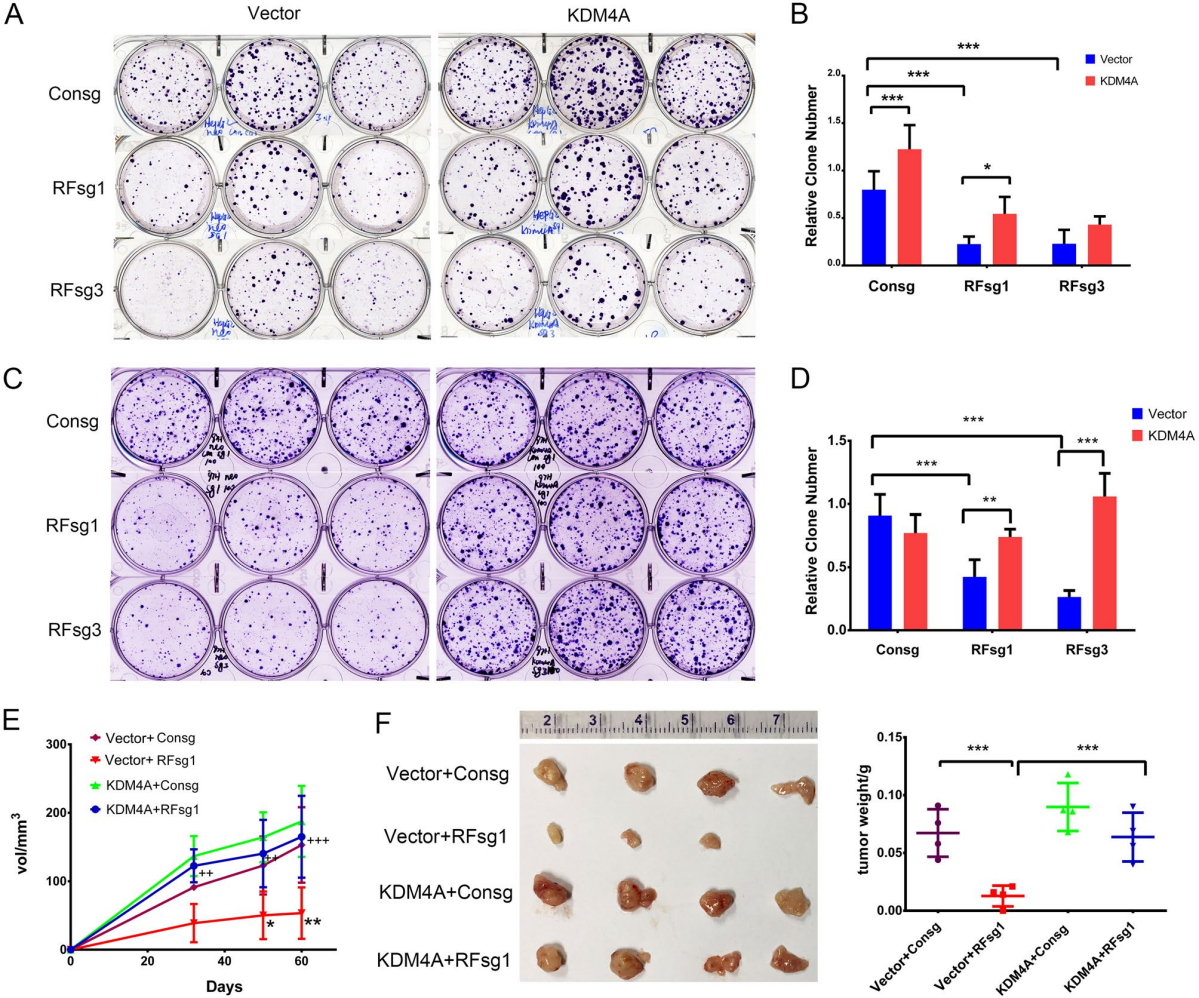
KDM4A overexpression reverses the growth inhibitory effects of RFX5 silencing in HCC. (**A**,**B**) Clonogenicity assay of RFX5 sgRNAs (RFsg1, RFsg3)-transfected HepG2 cells and rescued with retroviral vector or retroviral expressing KDM4A vector (**A**), and clone number quantification derived from triplicate experiments (**B**). (**C**,**D**) Clonogenicity assay of RFX5 sgRNAs (RFsg1, RFsg3)-transfected MHCC-97H cells and rescued with retroviral Consg vector or retroviral expressing KDM4A vector (**C**), and the quantification of clone numbers from three independent experiments (**D**). (**E**,**F**) MHCC-97H cells, which were modified using lentivirus expressing espCas9 and sgRNAs against non-targeting control (Consg) or RFX5 (RFsg1) alone, and then transduced with retroviral KDM4A, were subcutaneously implanted in BALB/c Nude mice (n = 4 per group). Tumor volumes were determined via calipers (**E**). Extraction and weighing of tumors were done 60 days after MHCC-97H implantation (**F**). *P < 0.05, **P < 0.01, ***P < 0.001.

As KDM4A was closely regulated by RFX5 and correlated to the prognosis in HCC, we thus investigated whether KDM4A was functionally involved in the downstream pathway of RFX5 in HCC development. We firstly performed rescue experiments with the colony formation assay. The ability of MHCC-97H and HepG2 cells to form colonies were greatly compromised after knockdown of RFX5 with CRISPR/Cas9. However, overexpression of KDM4A in RFX5 depleted cells recovered the colony numbers (Fig. 4A–D).

We then performed rescue experiments in the tumor xenograft assay. Knockdown of RFX5 with CRISPR/Cas9 largely inhibited the subcutaneous growth of HCC cells in nude mice. However, overexpression of KDM4A in RFX5 depleted cells significantly recovered the tumor growth potential that was compromised by RFX5 knockdown (Fig. 4E,F). These results revealed that KDM4A overexpression could reverse the tumor suppressive effect of RFX5 silencing in HCC cells, indicating that KDM4A is a downstream effector of RFX5.

### RFX5-KDM4A pathway suppresses apoptosis and promotes cell cycle transition in HCC

As KDM4A has been reported to be an intrinsic factor involved in both apoptosis and cell cycle regulation^14–16^, we further determined the impact of RFX5-KDM4A pathway on apoptosis and cell cycle transition in HCC.

**Figure 5:**
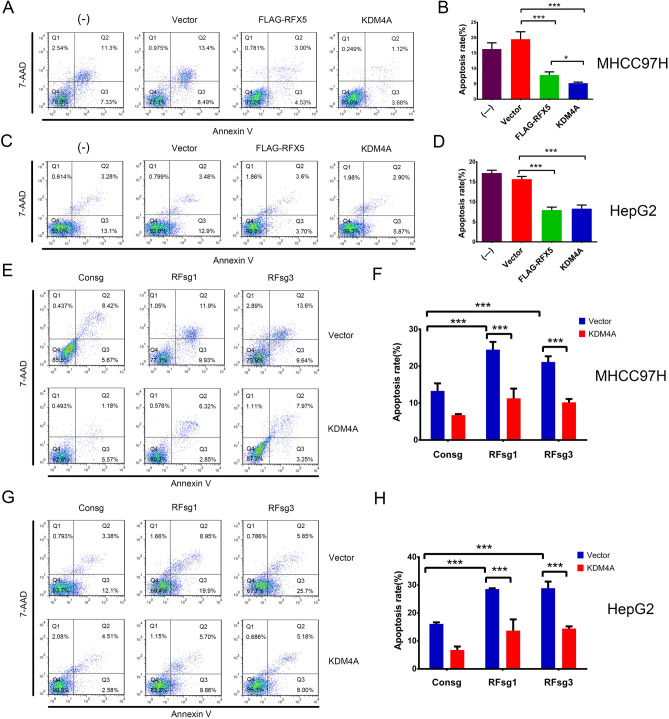
RFX5-KDM4A pathway negatively regulates apoptosis in HCC. HCC cells with different treatment were subjected to FCAS analysis with Annexin-V and 7AAD staining to quantify the apoptotic cells. The frequency of Annexin-V positive cells was indicated as apoptotic cell population. (**A**–**D**) MHCC-97H (**A**,**B**) and HepG2 (**C**,**D**) cells transduced with a retroviral vector control, FLAG-RFX5 or KDM4A were treated with 5 nM ActD and subjected to apoptosis analysis (**A**,**C**) and one-way ANOVA (**B**,**D**). (**E**–**H**) MHCC-97H (**E**,**F**) and HepG2 (**G**,**H**) cells transduced lentiviral sgRNA targeting RFX5 together with retroviral control vector or KDM4A were treated with 5 nM ActD and subjected to apoptosis analysis (**E**,**G**) and one-way ANOVA (**F**,**H**). *P < 0.05, ***P < 0.001.

Firstly, we found that overexpression of RFX5 or KDM4A significantly suppressed Actinomycin D (ActD) induced apoptosis in both MHCC-97H (Fig. 5A,B) and HepG2 (Fig. 5C,D). However, RFX5 silencing with RFX5 sgRNA (RFsg1 or RFsg3) elevated apoptosis in both MHCC-97H (Fig. 5E,F) and HepG2 (Fig. 5G,H).

To determine if RFX5 was protective against KDM4A-mediated apoptosis in HCC cells, we artificially overexpressed KDM4A in RFX5-depleted HCC cells and subjected them to apoptosis assay. As expected, KDM4A overexpression significantly inhibited RFX5 silencing-mediated cellular apoptosis in both MHCC-97H (Fig. 5E,F) and HepG2 (Fig. 5G,H). These results revealed that RFX5-KDM4A pathway could prevent HCC cells from apoptosis.

**Figure 6:**
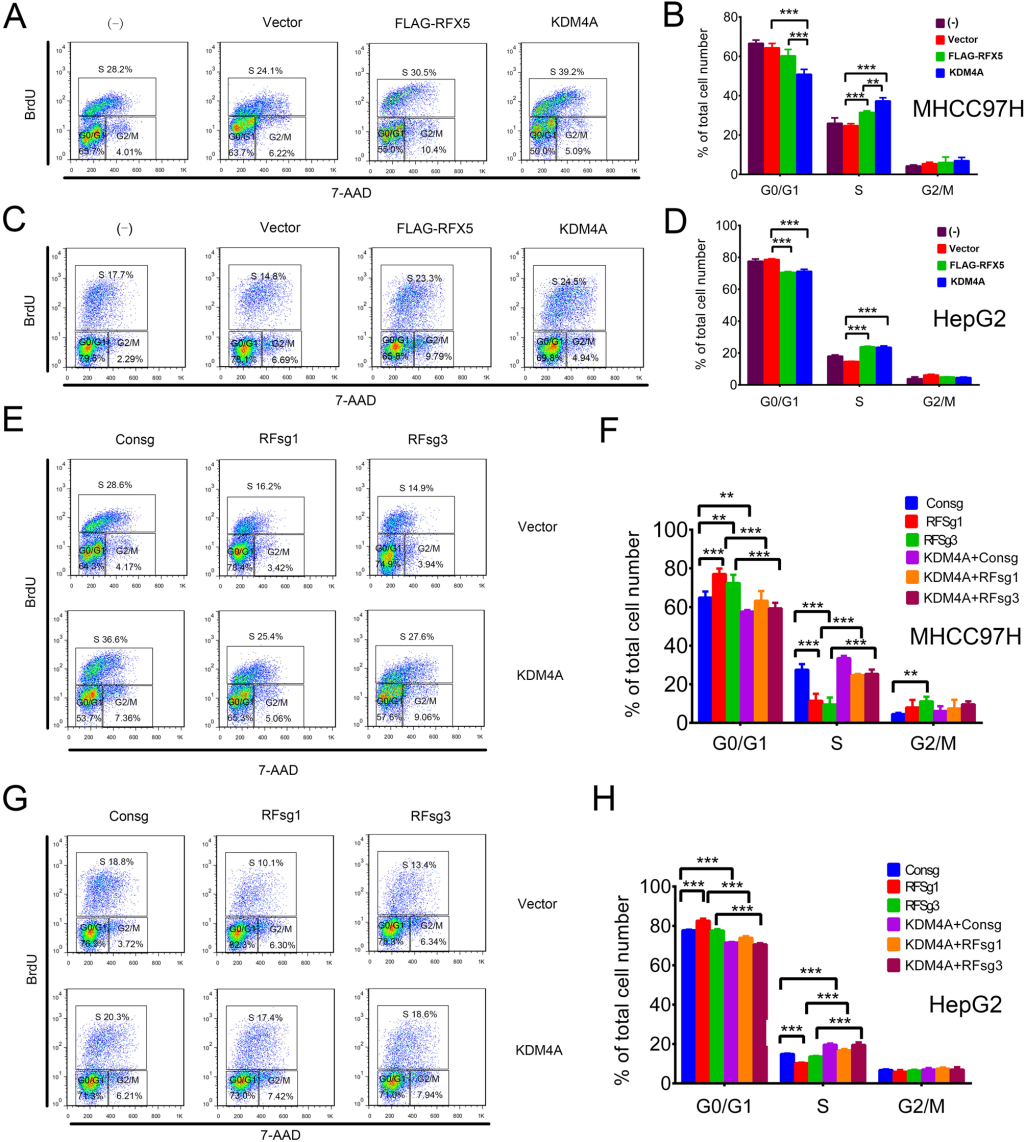
RFX5-KDM4A pathway promotes cell cycle transition in HCC. HCC cells with different treatment were subjected to FACS analysis with anti-BrdU and 7AAD staining to measure the cell cycle transition. (**A**–**D**) MHCC-97H (**A**,**B**) and HepG2 (**C**,**D**) cells transduced with the control lentiviral vector, FLAG-RFX5 or KDM4A were subjected to cell cycle analysis (**A**,**C**) and One-way ANOVA analysis (**B**,**D**). (**E**–**H**) MHCC-97H (**E**,**F**) and HepG2 (**G**,**H**) cells transduced with lentiviral sgRNA targeting RFX5 together with the control retroviral vector or KDM4A were subjected to cell cycle analysis (**E**,**G**) and One-way ANOVA analysis (**F**,**H**). **P < 0.01, ***P < 0.001.

Moreover, to examine whether RFX5 was a key factor in promoting HCC cells proliferation, 7-AAD and BrdU staining was performed to examine the proportion of HCC cells in each cell cycle. HCC cells were first serum-starved for 3 days in order to synchronize them at the G1 phase. Intriguingly, overexpression of RFX5 or KDM4A promoted G1/S phase transition in MHCC-97H (Fig. 6A,B) and HepG2 (Fig. 6C,D). The percentage of S phase cells was markedly higher in RFX5-transfected MHCC-97H (31.23 ± 0.71%) and HepG2 cells (23.87 ± 0.35%) than that in vector-transfected MHCC-97H (24.7 ± 1.01%, P < 0.001) and HepG2 cells (14.1 ± 0.1%, P < 0.001). Meanwhile, the percentage of G1 phase cells was markedly lower in RFX5-transfected MHCC-97H and HepG2 cells than that in vector-transfected MHCC-97H and HepG2 cells. However, the G2/M proportion was only moderately affected.

Next, we assessed the impact of RFX5 knockdown on cell cycle transition. We found that cell numbers at G1 phase were significantly increased in both MHCC-97H and HepG2 cells with RFX5 knockdown, while the converse was seen for the S phase cell numbers (Fig. 6E–H).

We also showed that elevated expressions of KDM4A were able to ablate the RFX5-mediated cell cycle transition inhibitory effects in RFX5 depleted-MHCC-97H and HepG2 cells. In contrast to cells transfected with RFsg (RFsg1 or RFsg3), the cell population in G1 phase was significant decreased when MHCC-97H and HepG2 cells were co-transfected with KDM4A and RFsg. Meanwhile, the cell proportion in S phase was largely increased in cells co-transfected with KDM4A and RFsg (Fig. 6E–H).

### RFX5 negatively regulates p53 signaling pathway through KDM4A

In previous study, we had proved that RFX5 could inhibit DNA damage-induced apoptosis through regulating the p53-Bax signaling pathway^7^. The transcriptional activation of p21 (also known as protein CDKN1A) in response to damaged DNA was mainly dependent on p53 and further played a vital role in arresting cell proliferation^5^. In efforts to further uncover the biological pathways behind RFX5-regulated cell cycle progression and apoptosis, the expressions of KDM4A, p53, p21 and Bax were quantified in HCC cells using Western blot analysis.

**Figure 7:**
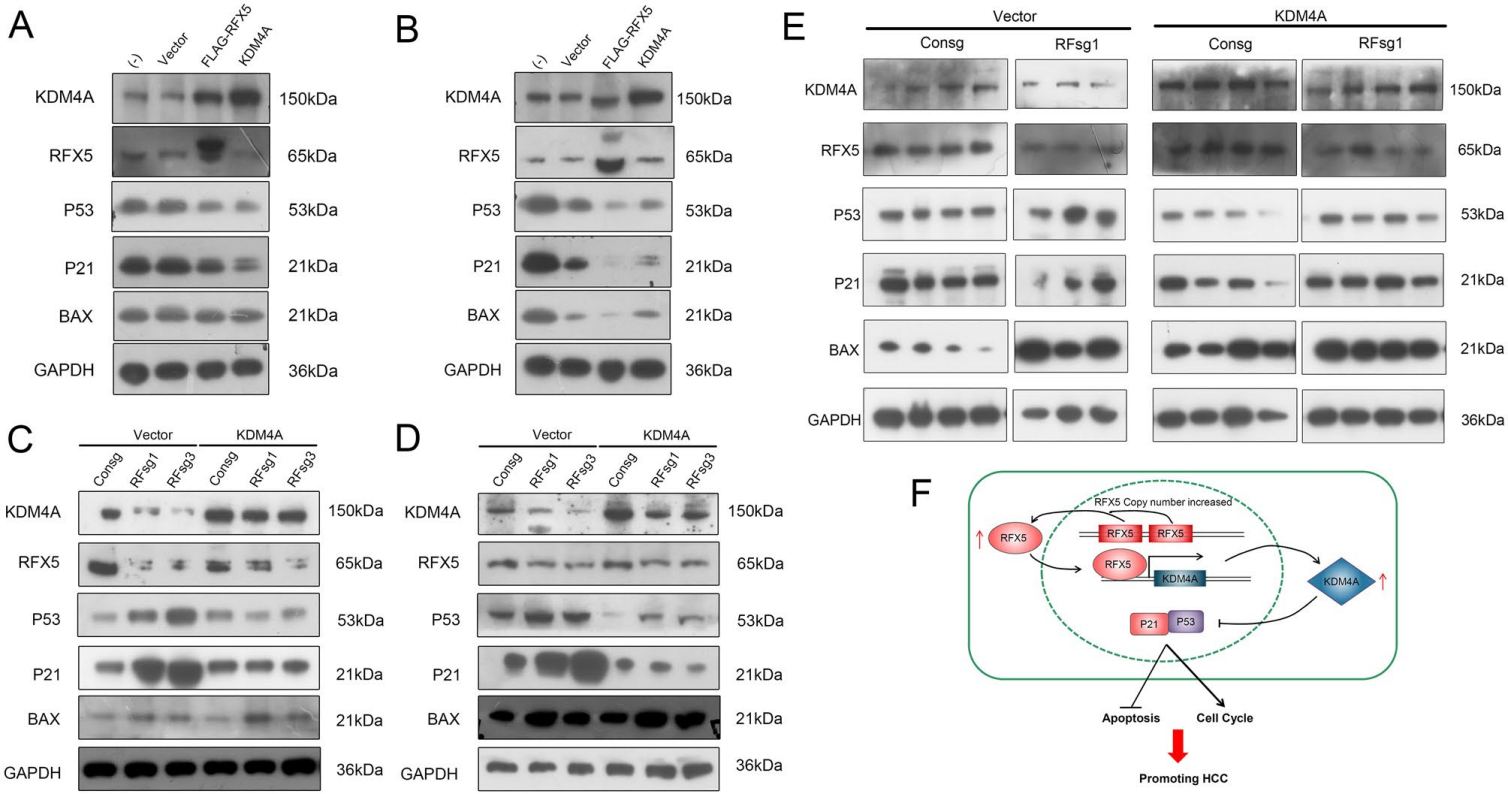
RFX5-KDM4A negatively regulates p53 and downstream pathway. (**A**,**B**) Total proteins from MHCC-97H (**A**) and HepG2 (**B**) cells transduced with either KDM4A, FLAG-RFX5 or lentiviral vector control were determined with Western blot. (**C**,**D**) Total proteins from MHCC-97H (**C**) and HepG2 (**D**) cells transduced with lentiviral sgRNA targeting RFX5 together with either retroviral control vector or KDM4A were determined with Western blot. (**E**) Total proteins from xenograft tumors were determined with Western blot analysis. (**F**) The mechanism underlying RFX5 and KDM4A in HCC progression.

We found that RFX5 overexpression up-regulated KDM4A protein levels, which confirmed that RFX5 could positively regulated the expression of KDM4A in HCC cells. Interestingly, we also found that raised RFX5 levels significantly suppressed the protein expressions of p53, p21 and Bax in both MHCC-97H and HepG2 (Fig. 7A,B). Conversely, RFX5 silencing dramatically suppressed KDM4A protein levels, but up-regulated p53, p21 and Bax proteins in both MHCC-97H and HepG2 (Fig. 7C,D). These results indicated that RFX5 directly regulates p53-signaling pathway.

It has been identified that KDM4A could promote colon carcinoma proliferation via suppression of the p53-p21 signaling pathway^14^. We thus investigated whether RFX5 negatively regulate p53 signaling pathway through KDM4A. Indeed, overexpression of KDM4A decreased p53 and p21 protein levels in both MHCC-97H and HepG2, which was consistent with the result observed after RFX5 overexpression. However, the protein level of Bax was not affected (Fig. 7A,B). Notably, overexpressing KDM4A in RFX5-depleted HCC cells decreased p53 and p21 protein levels to similar level in parent MHCC-97H and HepG2 cells. However, overexpressing KDM4A failed to decrease the protein level of Bax in RFX5-depleted cells (Fig. 7C,D).

In mice xenograft tumor samples, we also found similar results that knockdown of RFX5 upregulated p53 and p21 protein expressions, while overexpression of KDM4A in RFX5-depleted HCC cells decreased p53 and p21 protein expressions to similar levels in their parent cells. However, the quantity of Bax proteins remained unaffected (Fig. 7E).

Our results thereby identified that KDM4A represents an additional mechanism through which RFX5 suppresses the p53 signaling pathway and promotes hepatocellular carcinoma progression (Fig. 7F).

## Discussion

RFX5 is a transcription factor encoding DNA binding protein and classically functions as a transcription regulator of crucial MHCII gene. It is involved in the development of bare lymphocyte syndrome (BLS), a severe immunodeficiency disease^17^. Our previous study confirms that RFX5 regulated non-MHCII target genes as a putative tumor driver gene to promote HCC development^10^. Notably, RFX5 overexpression is an independent prognostic factor for the survival of patients with advanced HCC. Through systematical identification of transcriptional target genes of RFX5, it was revealed that RFX5 might also be involved in cell cycle, DNA damage repair and proliferation pathways. The current study reveals KDM4A, a Histone demethylase, to be a potential down-stream gene of RFX5 in the pathogenesis of HCC. KDM4A was found to be under tight control of RFX5-mediated regulation in both HCC cell lines and tissues.

In this study, we provided solid evidence that KDM4A was closely involved in the development of HCC. KDM4A protein and mRNA levels strongly correlated to HCC patient prognosis. We also showed that the up-regulation of KDM4A promoted HCC proliferation and suppressed apoptosis. Notably, KDM4A overexpression was able to reverse the pro-apoptotic, cell cycle transition inhibitory, and growth inhibitory effects of RFX5 depletion in HCC cells, highlighting KDM4A is a dominant downstream effector of RFX5.

KDM4A, also named the lysine-specific demethylase 4A (KDM4A), belongs to a demethylase subfamily known as the JMJD2 family, which consists of the 4 homologous proteins (JMJD2A, JMJD2B, JMJD2C, and JMJD2D)^18^. Histone demethylation involves modification of chromatin formation and is a crucial component in the construction of protein complexes controlling gene expression. Histone demethylation enzymes have been proved to be frequently involved in tumor development^12^. Previous study has reported that KDM4A could promote malignant proliferation of human liver cancer cell line Hep3B, which was consistent with our findings^13^.

Previous publications have revealed that KDM4A could regulate apoptosis, cell cycle and cell proliferation through mediation of p53 degradation and suppression of p21^14,15,19^. Our observation was consistent with previous reports that KDM4A overexpression was associated with reduced p53 and p21 levels, while exerting only a minor impact on Bax protein levels. However, in previous study, we identified that RFX5 negatively regulated p53 and Bax via transactivation of YWHAQ^7^.

In addition, the colony formation assay demonstrated that KDM4A, not YWHAQ (Supplementary Fig. 1B), was able to reverse the inhibitory effect of RFX5 silencing in MHCC-97H cells. Conversely, YWHAQ^7^, not KDM4A (Supplementary Fig. 1A), could rescue inhibition of colony formation that was induced by RFX5 knockdown in SK-HEP-1 cells. However, KDM4A and YWHAQ^7^ could both partly rescue inhibition of colony formation induced by RFX5 depletion in HepG2. Collectively, it suggested that RFX5 could regulate both the p53-p21 and p53-Bax pathways in a cellular environment-dependent manner.

KDM4A has been reported to be involved in the development and prognosis of several tumors, including bladder cancer^20^, breast cancer^21^, colon cancer^14^, lung cancer^22^, etc.^23–26^. The current investigation establishes the relationship between RFX5 and KDM4A in HCC. Taking into consideration that there was a significant correlation between RFX5 and KDM4A which were both overexpressed in a wide range of tumors based on TCGA data (Supplementary Fig. 2S), more extensive studies of the RFX5-KDM4A pathway in other tumors should be conducted. Moreover, specific inhibitors that target the JmjC histone demethylases (KDMs) have been developed and are undergoing evaluation^27–30^, a promising avenue in the development of more efficient treatment to HCC.

With the exception of KDM4A, several candidates among the RFX5 downstream genes were found to be involved in cancer-related pathways such as cell cycle proliferation, DNA damage repair and proliferation pathways, including several well-known cancer driver genes, such as CCNB1^31^, PCNA^32^ and BRCA1^33,34^. The biological role of these genes in the RFX5 downstream pathway and in HCC should also be explored in the future.

However, there were some limitations in our study. First, we need to collect more HCC samples to prove the relationship between the RFX5 expression and the prognosis of patients with early and intermediate-stage HCC. Second, KDM4A mainly functions as an enzyme that catalyzed demethylation of H3K9me3 and H3K36me3^14^. It has been proved that KDM4A could be recruited together with p53 to the promoter of p21 and down-regulated the level of H3K9me_3_ at p21 in colon cancer cells. In particular, Ann et al. discovered KDM4A could inhibit P21 expression by reducing H3K9me3 expression in human liver cancer line Hep3B^13^. The mechanism of KDM4A in HCC should be conducted in further studies.

In summary, RFX5 transcriptionally activates KDM4A expression, which drives hepatocellular carcinoma progression by inhibiting apoptosis, accelerating cell cycle transition and subsequently promoting HCC development.

## Methods

### RNA sequencing data and ChIP-seq data

RNA Sequencing (RNA-seq) data was acquired from the liver hepatocellular carcinoma (LIHC) database of the Cancer Genome Atlas (TCGA) project. ChIP-seq data was obtained from the HepG2 database of the Encyclopedia of DNA Elements (ENCODE) project. Analysis and visualization of data were carried out as described in previous reports^11^. The survival analysis in TCGA LIHC cohort was determined by Kaplan–Meier plotter browser (https://kmplot.com/analysis/index).

### HCC tissue samples and cell lines

Sampling of human HCC specimens (tumor and adjacent non-cancerous tissues) was performed promptly after hepatectomies done at the affiliated hospital of Guilin Medical University (Guilin cohort). Prior to collection, all patients were counselled regarding the use of their tissues for this study and provided written consent. Experimental protocols were approved by the Ethics Committee of Peking University People's Hospital and the Affiliated Hospital of Guilin Medical University. HepG2, SK-HEP-1 and HEK293T cells were obtained from the American Type Culture Collection (ATCC, USA) and maintained in Eagle's Minimum Essential Medium (ATCC). MHCC-97H cells were kindly provided by the Academy of Military Medical Science (Beijing, China) and maintained in Dulbecco’s modified Eagle’s medium (Gibco). GP2-293 cells were procured from the Cell Bank of the Chinese Academy of Sciences (Shanghai, China) and grown in Dulbecco’s modified Eagle’s medium. All media were enriched with penicillin–streptomycin antibiotics (Gibco) and 10% fetal bovine serum. The cells were cultured at 5% CO_2_ at 37 °C.

### Plasmid constructions, retroviral and lentiviral productions and stable cell lines establishment

The pGL4-KDM4A luciferase reporter vector was built through insertion of an active KDM4A promoter region tightly bound by RFX5 into the pGL4.10 vector (Promega, Madison, WI, USA). The sequence of the open reading frame (ORF) of Human KDM4A was cloned into the retroviral vector pMSCVneo, with the final product named pMSCV-KDM4A. Lentiviral vector expressing FLAG-tagged RFX5 ORF (FLAG-RFX5), SgRNA (RFsg1 and RFsg3) and shRNA (RFK1 and RFK2) specifically targeting human RFX5 were cloned as documented in previous reports^7^. Both lentiviral and retroviral construction, infection, purification and production of stable cell lines were carried out as per previously documented protocols^7^. The effect of RFX5 knockdown induced by RFsg1 or RFsg3 in HepG2 cells was detected by Sanger sequencing (Supplementary Fig. 3).

### ChIP and luciferase assays

The ChIP assay was carried out in compliance to manufacturer protocols (Millipore, Billerica, MA, USA). The DNA Fragments from SK-HEP-1, MHCC-97H and HepG2 were incubated with normal rabbit IgG or rabbit anti-human RFX5 antibody (Rockland, Gilbertsville, PA, USA). The ChIP-PCR analysis was applied to detect the purified free DNA fragments with primers covering KDM4A promoter sequences (KDM4A-PF: ACCCTTGAATTGGTTGACTC; KDM4A-PR: GGCCGATCCTACTGCTTT). The dual luciferase activity assay of HCC cells co-transfected with RFX5 expression plasmid and firefly luciferase plasmid was performed according to our previous procedure^7^.

### Tissue microarray and immunohistochemistry

The tissue microarray (TMA) containing 128 pairs of matched primary HCC tumor and adjacent tissue samples derived from the Guilin cohort was used for immunohistochemistry (IHC) staining. TMA slides were subjected to antigen retrieval in EDTA buffer (pH 8.0) for 15 min at 100 °C. Rabbit anti-human KDM4A antibody (Cell Signaling Technology, #5328S; 1:25 dilution) or rabbit anti-human RFX5 antibody (Novus Biologicals, #NBP1-86041; 1:100 dilution) were incubated with the cell samples overnight at 4 °C. Expressions of RFX5 and KDM4A protein were quantified independently by two pathologists based on the total percentage of nuclear-staining positive tumor cells. A detailed assay on IHC staining along with the scoring standard of RFX5 and KDM4A protein expression was carried out as our previous report^7^.

### Immunofluorescence staining

Multiplex IHC staining was performed on the slices of formalin-fixed paraffin-embedded (FFPE) tumor tissue samples using an opal 4-color manual IHC Kit (NEL810001KT, PerkinElmer) according to the manufacturer’s instructions. Briefly, after epitope retrieval, samples were blocked for 15 min at 37 °C and then incubated overnight at 4 °C with rabbit anti-human RFX5 antibody (Novus Biologicals, #NBP1-86041, 1:300; dilution) and rabbit anti-human KDM4A antibody (Abcam, #ab191433, 1:100; dilution). After incubating with Polymer horseradish-peroxidase (HRP)-conjugated secondary antibody specific to rabbit (PerkinElmer) for 10 min at room temperature, slices were subjected to tyramide signal amplification (TSA) to generate Opal signal. Then, antibodies in slices were stripped after heating in microwave, and the slices were ready for staining the next target protein. After all target proteins were labeled, images were collected by Vectra Polaris automated quantitative pathology system (PerkinElmer).

### RNA extraction and quantitative real-time-PCR

TRIzol reagent (Invitrogen) was used for reverse transcription of total RNA into cDNA following protocols set by the manufacturer (Applied Biosystems). The quantitative real-time PCR (QRT-PCR) assay was carried out using a LightCycler 480 (Roche Applied Science). KDM4A primer sequences for QRT-PCR were used as follows: Forward, 5′-ACCCTTGAATTGGTTGACTC-3′ and Reverse, 5′-GGCCGATCCTACTGCTTT-3′. The primers of RFX5 and GAPDH were used as the same as previously reported^10^. GAPDH was used as the internal control gene in order to derive relative gene expressions. All results were the product of three separate experiments.

### In vivo tumorigenicity experiments

MHCC-97H cells were firstly infected by retrovirus expressing KDM4A or control vector, and then stably transduced with the CRISPR/Cas9 system with lentiviral expressing RFX5 sgRNAs (RFsg1) or sgRNA control (Consg). Four stable cell lines were acquired as follows: Vector + Consg group, Vector + RFsg1 group, KDM4A + Consg group and KDM4A + RFsg1 group. The stable cell lines were subcutaneously injected into the dorsal flank of BALB/c Nude mice (male, 4 weeks) at 2 × 10^6^ cells per mouse. After 3 weeks of observation, the resultant tumors were measured with vernier calipers. Tumor volumes were calculated as previously described^7^. Animal experiments were approved by the Ethics Committee of Peking University People’s Hospital.

### Clonogenicity assay

HCC stable cell lines generated by lentiviral or retroviral infection were subjected to clonogenicity assays. Single-cell suspensions from each were plated at a density of 1,000 cells per well in 6-well plates. All experiments were done in triplicate. Colonies that grew to a detectable size after two weeks were harvested and fixed with 4% paraformaldehyde for 10 min. All colonies were subjected to 30 min of staining with 0.05% crystal violet before being rinsed with distilled water and air-dried.

### Western blot analysis and antibody

Protease inhibitor cocktail (Roche)—supplemented RIPA buffer was used to lyse HCC cells. 10 μg of the protein lysates were used for protein detection. Proteins were electrophoresed using SDS/PAGE gels (Thermo Fisher Scientific), blotted onto membranes for analysis and incubated overnight at 4 °C with the following primary antibodies: RFX5 (Proteintech, #12137-1-AP; 1:1,000 dilution), KDM4A (Cell Signaling Technology, #5328S; 1:500 dilution), P21 (Cell Signaling Technology, #2947S; 1:1,000 dilution), P53 (Proteintech, #104421-1-AP; 1:2000 dilution), Bax (Proteintech, #50599-2-Ig; 1:2000 dilution), or GAPDH (Proteintech, #HRP‑60004; 1:5,000 dilution). Membranes were then rinsed thrice with 1X TBST and subjected to another hour of co-incubation with HRP-conjugated second antibodies (Proteintech, #SA00001-2; 1:10,000 dilution) at room temperature.

### Flow cytometry analysis

The Annexin V-FITC apoptosis Detection Kit (BD Pharmingen, 559763) was used to determine the rates of cellular apoptosis. All procedures were in compliance to manufacturer’s protocols. Harvested cells were rinsed with ice-cold PBS prior to being stained with7-amino-actinomycin D (7-AAD) and Annexin V-FITC. After incubation for 15 min in darkness, the cells were detected by FACSAria II (BD Biosciences). Apoptotic cells were those that stained positive for annexin-V.

Cell cycle kinetics and the degree of BrdU incorporation into the DNA of proliferative cells were assessed with a 5′-bromo-2′-deoxyuridine (BrdU) flow kit (BD Pharmingen, 552598), based on instructions stipulated by the manufacturer. All cells were first subjected to a 1 h incubation with 10 μM BrdU at 37 °C. Each treatment point contained three independent cell samples which were harvested and fixed in a Cytofix/Cytoperm buffer before being incubated for another hour with DNAase at 37 °C. Next was the addition of FITC-conjugated anti-BrdU antibody (1:50 dilution in Wash buffer), with the cells left to incubate at room temperature for 20 min. The washing buffer was used to rinse cells before they were stained with 7-AAD. Lastly, FACSAria II (BD Biosciences) was used to detect cells. Cell apoptosis and profiles of cell cycle stages were analyzed with FlowJo analysis software (Tree Star).

### Statistical analysis

The GraphPad Prism 7.0 (GraphPad Software, USA) and R Statistical Software (Foundation for Statistical Computing, Austria) was used for all data analysis. Each dataset is depicted in terms of mean ± standard error, with each result derived as a composite of a minimum of three different experiments. Comparison between two or more groups was carried out using the two-tailed Student’s *t* test or ANOVA. The Pearson Chi-square test and survival analyses were carried out by Kaplan–Meier plots and log-rank tests in order to determine clinically significant associations. P < 0.05 was considered to be statistically significant.

### Ethical approval

All methods were performed in accordance with the Declaration of Helsinki. This study was approved by the Ethics Committee of Peking University People's Hospital and the Affiliated Hospital of Guilin Medical University. Informed consent was obtained from all patients in this study.

## Supplementary information

Supplementary Information.
