# Supplementary material for: RFX5 promotes the progression of hepatocellular carcinoma through transcriptional activation of KDM4A
Source: Sci Rep. 2020 Sep 3;10:14538. doi: 10.1038/s41598-020-71403-1 (PMC7471945; doi:10.1038/s41598-020-71403-1)
Supplement: Supplementary file 1 — Supplementary Information. [file 41598_2020_71403_MOESM1_ESM.docx]

**Title: RFX5 promotes the progression of hepatocellular carcinoma through transcriptional activation of KDM4A**

Dong-Bo Chen^1, #^, Xing-Wang Xie^1, #^, Yang-Jing Zhao^2^, Xue-Yan Wang^1^, Wei-Jia Liao^3^, Pu Chen^3^, Kang-Jian Deng^3^, Ran Fei^1^, Wan-Ying Qin^3^, Jiang-Hua Wang^1^, Xu Wu^4^, Qi-Xiang Shao^2^, Lai Wei^1^, Hong-Song Chen^1, *^

^1^Peking University People’s Hospital, Peking University Hepatology Institute, Beijing Key Laboratory of Hepatitis C and Immunotherapy for Liver Disease, Beijing 100044, China

^2^Department of Immunology, and the Key Laboratory of Laboratory Medicine of Jiangsu Province, School of Medicine, Jiangsu University, Zhenjiang, Jiangsu 212013

^3^Laboratory of Hepatobiliary and Pancreatic Surgery, Affiliated Hospital of Guilin Medical University

^4^Center of Excellence, Becton Dickinson Biosciences, China Central Place, Beijing 100176, China

^#^The authors contributed equally to this work.

* To whom correspondence should be addressed: Hongsong Chen; Tel: +86 10 88325726; Fax: +86 10 88325723; Email: [chenhongsong@pkuph.edu.cn](mailto:chenhongsong@pkuph.edu.cn).

**Supplementary Table S1**

**Clinical characteristics of the patients included in analyses of KDM4A expression by IHC**

| **Clinical Parameters** | **Frequency (%)** | **KDM4A IHC staining (n=128)** | | |
| --- | --- | --- | --- | --- |
|  |  | **Negative**  **(n=86)** | **Positive (n=42)** | ***P ^b^*** |
| Gender | | | | |
| Male | 14 (10.9%) | 7 | 7 | 0.2502 |
| Female | 114 (89.1%) | 79 | 35 |  |
| Age^c^ | | | | |
| <55 | 89 (69.5%) | 56 | 33 | 0.1775 |
| ≥55 | 39 (30.5%) | 30 | 9 |  |
| Alcohol | | | | |
| No | 52 (40.6%) | 34 | 18 | 0.8668 |
| Yes | 76 (59.4%) | 52 | 24 |  |
| Tumor size, cm^c^ | | | | |
| <6 | 31 (24.4%) | 16 | 15 | 0.04718 |
| ≥6 | 96 (75.6%) | 70 | 26 |  |
| AFP, ng/mL | | | | |
| <20.0 | 36 (28.1%) | 22 | 14 | 0.4798 |
| ≥20.0 | 92 (71.9%) | 64 | 28 |  |
| HBsAg | | | | |
| Negative | 29 (22.7%) | 17 | 12 | 0.3338 |
| Positive | 98 (76.6%) | 69 | 29 |  |
| Cirrhosis | | | | |
| No | 20 (15.7%) | 14 | 6 | 0.9529 |
| Yes | 107(84.3%) | 71 | 36 |  |
| TNM stage | | | | |
| Early(I, II) | 36 (28.3%) | 23 | 13 | 0.7735 |
| Late(III,IV) | 91 (71.7%) | 63 | 29 |  |
| Multinodularity | | | | |
| No | 59 (46.1%) | 41 | 18 | 0.7455 |
| Yes | 69 (53.9%) | 45 | 24 |  |
| Lympho-invasion | | | | |
| No | 115 (89.8%) | 80 | 35 | 0.1638 |
| Yes | 13 (10.2%) | 6 | 7 |  |
| Venousinfiltration | | | | |
| No  Yes | 95 (74.2%) | 62 | 33 | 0.5676 |
|  | 33 (25.8%) | 24 | 9 |  |
| Metastasis & invasion | | | | |
| No | 100 (69.5%) | 60 | 29 | 1 |
| Yes | 39 (30.5%) | 26 | 13 |  |
| RFX5 | | | | |
| Negative | 37(28.9%) | 32 | 5 | 0.0058 |
| Positive | 91 (71.1%) | 54 | 37 |  |

**Supplementary Fig S1**

**
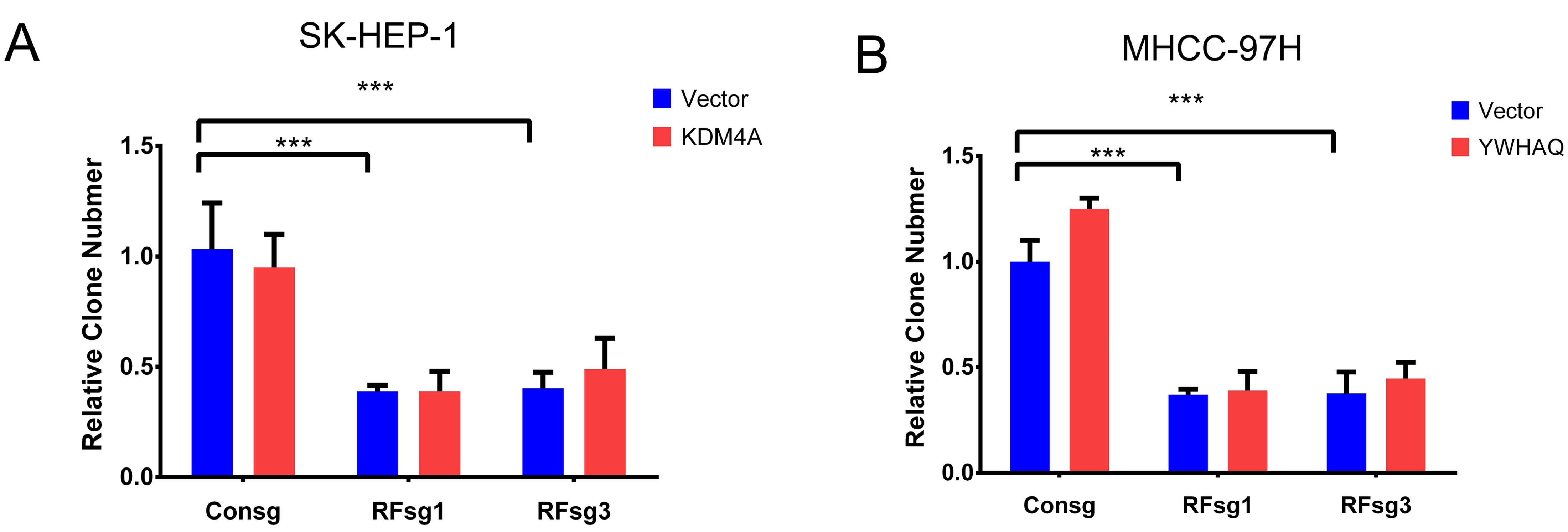
**

Supplementary Fig. S1. Overexpression of KDM4A or YWHAQ in RFX5 depleted cells could not recover the colony numbers in different liver cancer cell lines. (A) Clonogenicity assay of SK-HEP-1 cells infected with RFX5 sgRNAs (RFsg1, RFsg3) and rescued with retroviral vector or retroviral expressing KDM4A vector, and the quantification of clone numbers from three independent experiments. (B) Clonogenicity assay of MHCC-97H cells infected with RFX5 sgRNAs (RFsg1, RFsg3) and rescued with retroviral vector or retroviral expressing YWHAQ vector, and the quantification of clone numbers from three independent experiments. ***p < 0.001.

**Supplementary Fig S2**

**
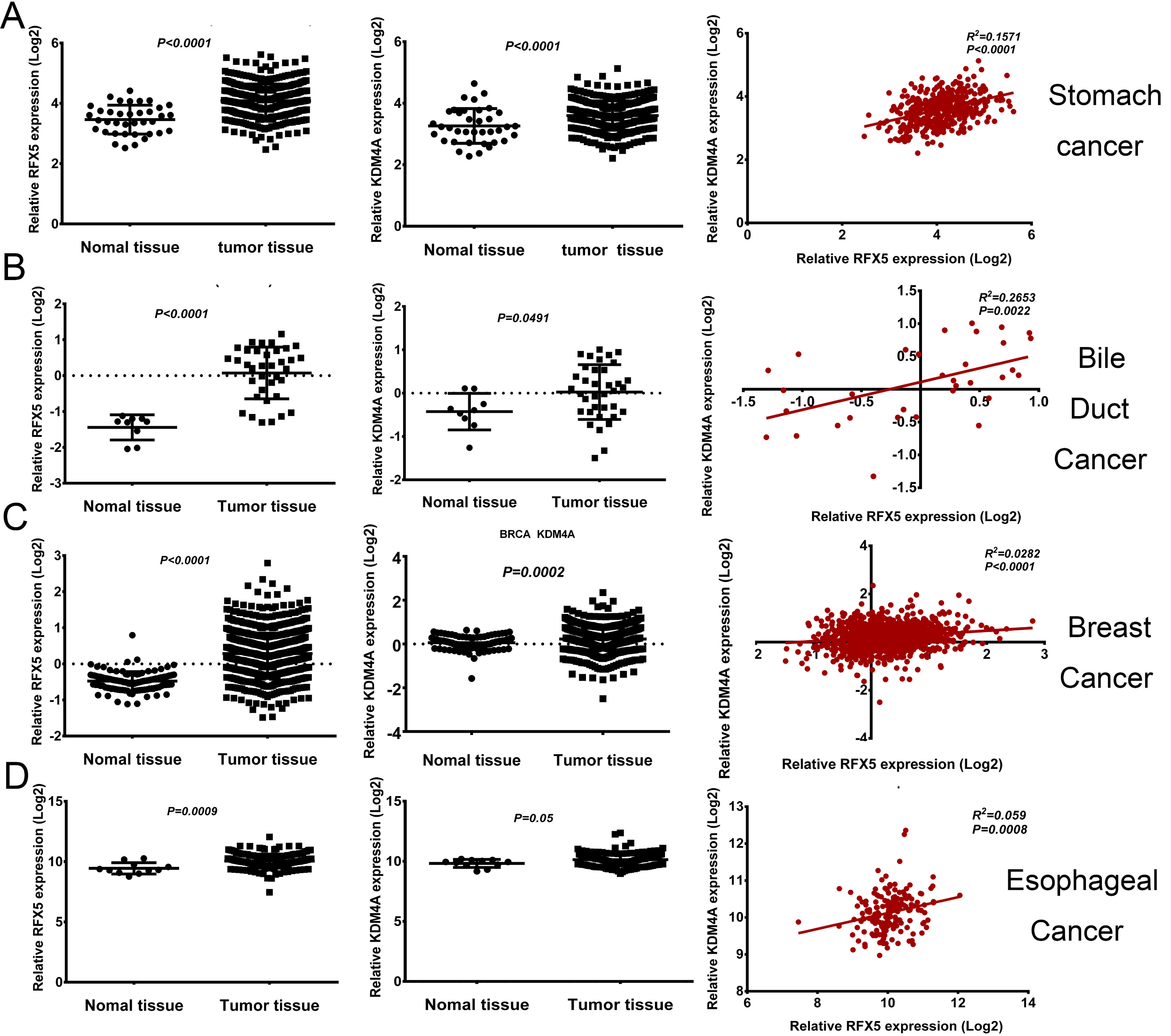
**

**
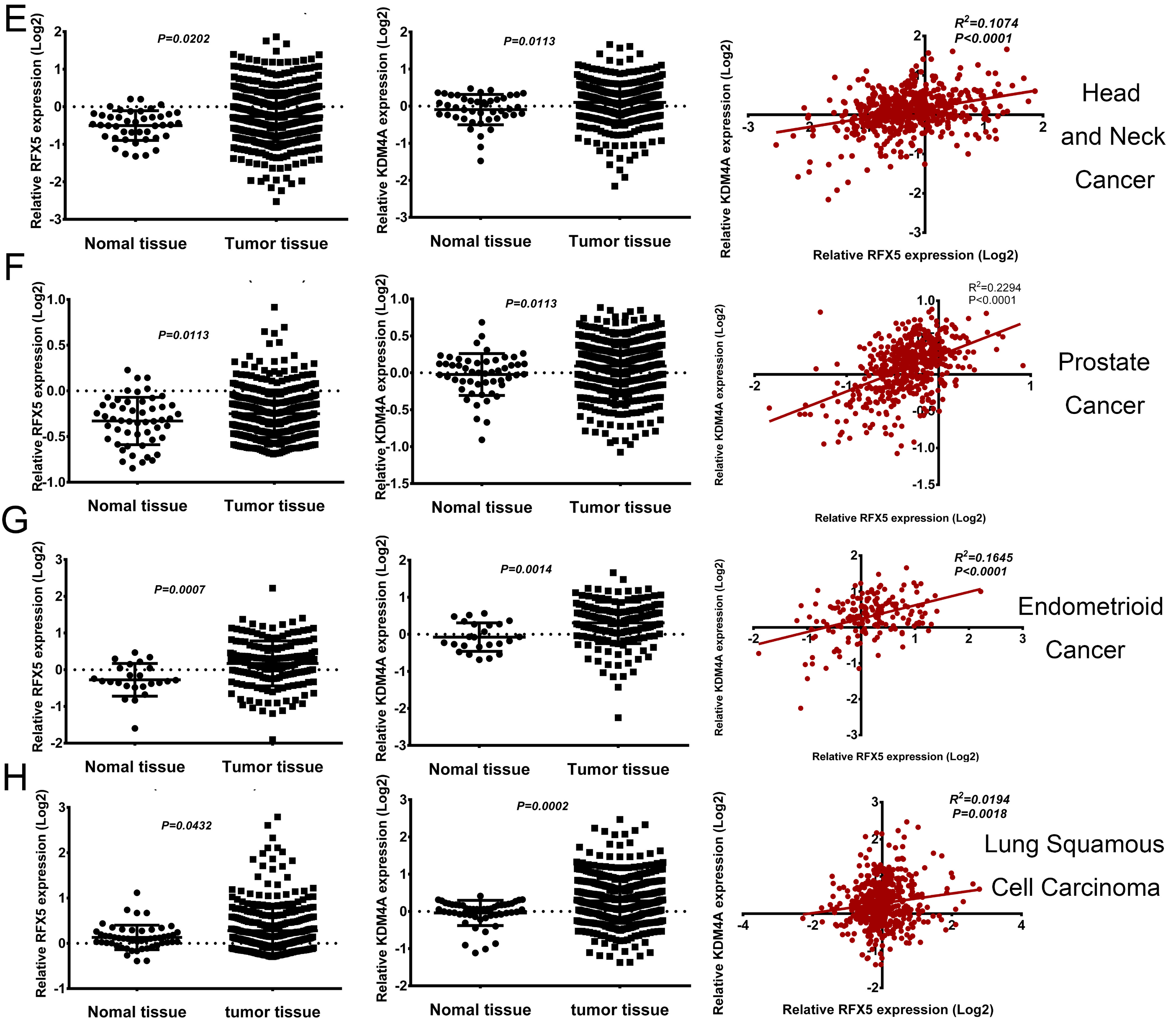
** **
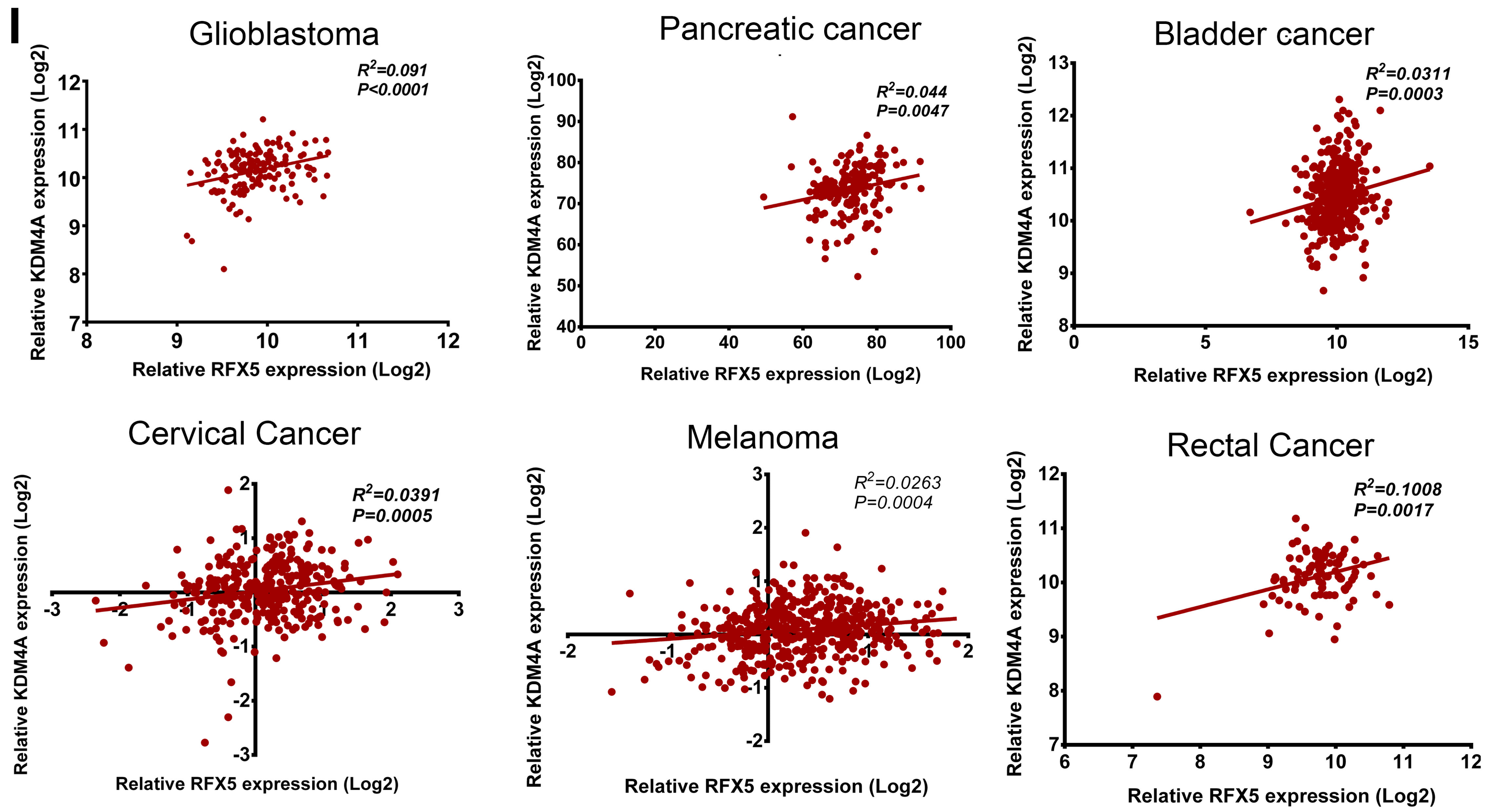
**

Supplementary Fig. S2. RFX5 and KDM4A were both overexpressed in a wide range of tumors based on TCGA data and there was a significant relationship between them. (A) The mRNA expressions of RFX5 and KDM4A in stomach cancer tissues were determined by RNA-seq data from TCGA dataset. The correlation between the mRNA expressions of RFX5 and KDM4A in patients with stomach cancer from TCGA dataset was analyzed. (B) The mRNA expressions of RFX5 and KDM4A in bile duct cancer tissues were determined by RNA-seq data from TCGA dataset. The correlation between the mRNA expressions of RFX5 and KDM4A in patients with bile duct cancer from TCGA dataset was analyzed. (C) The mRNA expressions of RFX5 and KDM4A in breast cancer tissues were determined by RNA-seq data from TCGA dataset. The correlation between the mRNA expressions of RFX5 and KDM4A in patients with breast cancer from TCGA dataset was analyzed. (D) The mRNA expressions of RFX5 and KDM4A in esophageal cancer tissues were determined by RNA-seq data from TCGA dataset. The correlation between the mRNA expressions of RFX5 and KDM4A in patients with esophageal cancer from TCGA dataset was analyzed. (E) The mRNA expressions of RFX5 and KDM4A in head and neck cancer tissues were determined by RNA-seq data from TCGA dataset. The correlation between the mRNA expressions of RFX5 and KDM4A in patients with head and neck cancer from TCGA dataset was analyzed. (F) The mRNA expressions of RFX5 and KDM4A in prostate cancer tissues were determined by RNA-seq data from TCGA dataset. The correlation between the mRNA expressions of RFX5 and KDM4A in patients with prostate cancer from TCGA dataset was analyzed. (G) The mRNA expressions of RFX5 and KDM4A in endometrioid cancer tissues were determined by RNA-seq data from TCGA dataset. The correlation between the mRNA expressions of RFX5 and KDM4A in patients with endometrioid cancer from TCGA dataset was analyzed. (H) The mRNA expressions of RFX5 and KDM4A in lung squamous cell carcinoma tissues were determined by RNA-seq data from TCGA dataset. The correlation between the mRNA expressions of RFX5 and KDM4A in patients with lung squamous cell carcinoma from TCGA dataset was analyzed. (I) The correlation between the mRNA expressions of RFX5 and KDM4A was analyzed in glioblastoma tissues, pancreatic cancer tissues, bladder cancer tissues, cervical cancer tissues, melanoma tissues, and rectal cancer tissues determined by RNA-seq data from TCGA dataset. *P<0.05* was considered statistically significant.

RNA Sequencing (RNA-seq) data in the Cancer Genome Atlas (TCGA) project (http://cancergenome.nih.gov) were obtained from the data hub of the UCSC Xena browser (http://xena.ucsc.edu/). The RNA-seq was performed using an Illumina HiSeq 2000 RNA Sequencing platform and then the gene-level TPM (transcripts per million) estimates was calculated with RSEM methods and log2(x+1) transformed.

**Supplementary Fig S3**


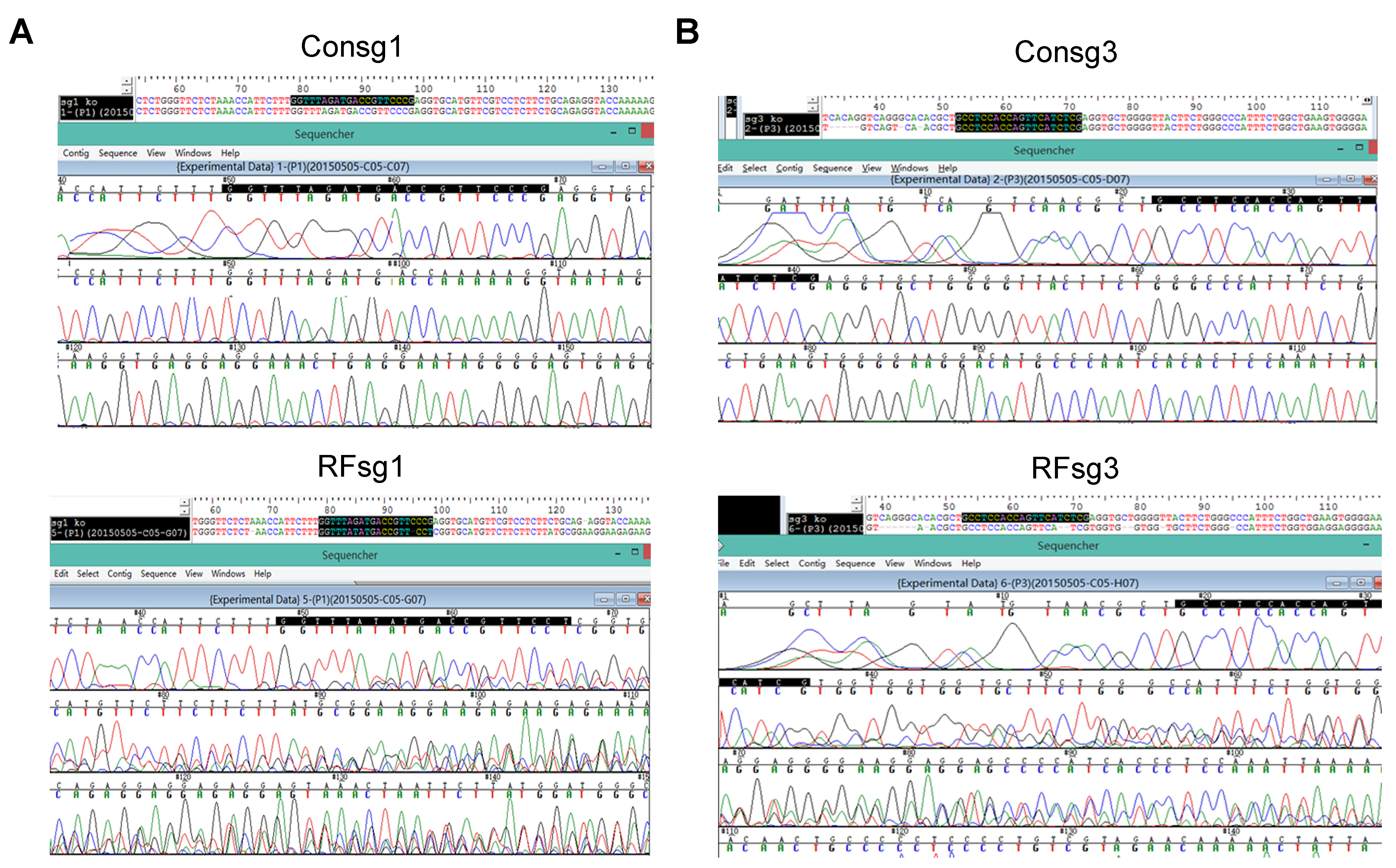


Supplementary Fig S3. RFX5 is knocked down by CRISPR/Cas9 system (RFsg1 and RFsg3) in HepG2 cells. A. The effect of RFX5 knockdown induced by Consg1 or RFsg1 in HepG2 cells was detected by Sanger sequencing. B. The effect of RFX5 knockdown induced by Consg3 or RFsg3 in HepG2 cells was detected by Sanger sequencing.

**The original gels/blots**


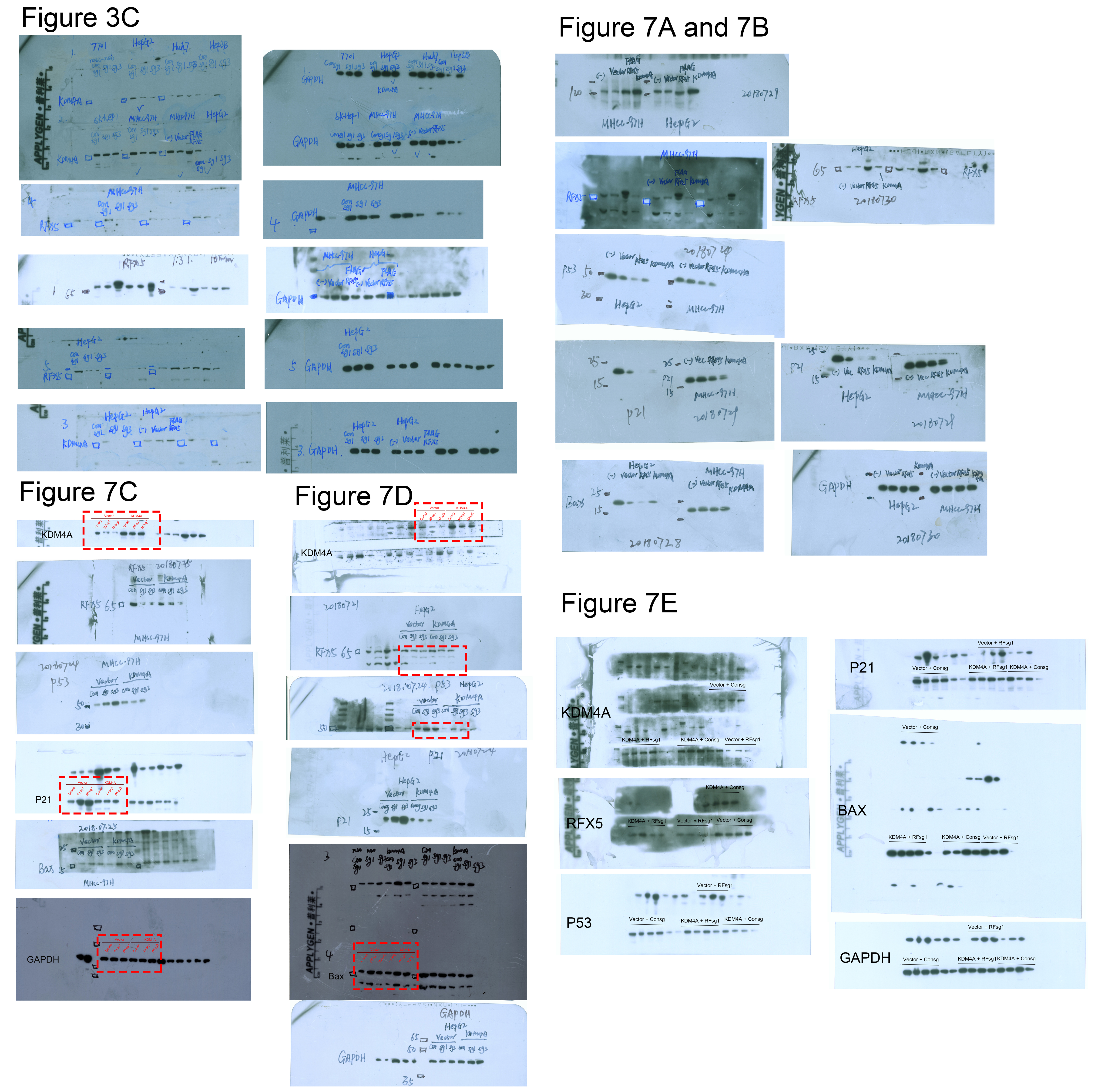


**Figure 3. RFX5 is a positive regulator of KDM4A expression in HCC**

C. KDM4A protein expression levels were determined in MHCC-97H and HepG2 cells that were transduced with lentiviral FLAG-RFX5 or sgRNA targeting RFX5 via Western blot analysis.

**Fig 7. RFX5-KDM4A negatively regulates p53 and downstream pathway**

A, B. Total proteins from MHCC-97H (A) and HepG2 (B) cells transduced with either KDM4A, FLAG-RFX5 or lentiviral vector control were determined with western blot. C, D. Total proteins from MHCC-97H (C) and HepG2 (D) cells transduced with lentiviral sgRNA targeting RFX5 together with either retroviral control vector or KDM4A were determined with western blot. E. Total proteins from xenograft tumors were determined with western blot analysis.
